# The nanofluidic confinement apparatus: studying confinement-dependent nanoparticle behavior and diffusion

**DOI:** 10.3762/bjnano.9.30

**Published:** 2018-01-26

**Authors:** Stefan Fringes, Felix Holzner, Armin W Knoll

**Affiliations:** 1 IBM Research - Zurich, Säumerstr. 4, 8803 Rüschlikon, Switzerland; 2 Department of Chemistry, University of Zurich, Winterthurerstrasse 190, CH 8057 Zürich, Switzerland; 3 SwissLitho AG, Technoparkstrasse 1, 8005 Zurich, Switzerland.

**Keywords:** Au nanospheres, confinement, nanofluidics, subdiffusion

## Abstract

The behavior of nanoparticles under nanofluidic confinement depends strongly on their distance to the confining walls; however, a measurement in which the gap distance is varied is challenging. Here, we present a versatile setup for investigating the behavior of nanoparticles as a function of the gap distance, which is controlled to the nanometer. The setup is designed as an open system that operates with a small amount of dispersion of ≈20 μL, permits the use of coated and patterned samples and allows high-numerical-aperture microscopy access. Using the tool, we measure the vertical position (termed height) and the lateral diffusion of 60 nm, charged, Au nanospheres as a function of confinement between a glass surface and a polymer surface. Interferometric scattering detection provides an effective particle illumination time of less than 30 μs, which results in lateral and vertical position detection accuracy ≈10 nm for diffusing particles. We found the height of the particles to be consistently above that of the gap center, corresponding to a higher charge on the polymer substrate. In terms of diffusion, we found a strong monotonic decay of the diffusion constant with decreasing gap distance. This result cannot be explained by hydrodynamic effects, including the asymmetric vertical position of the particles in the gap. Instead we attribute it to an electroviscous effect. For strong confinement of less than 120 nm gap distance, we detect the onset of subdiffusion, which can be correlated to the motion of the particles along high-gap-distance paths.

## Introduction

A fundamental understanding of the motion of micrometer- and nanometer-scaled objects in nanofluidic confinement is important for many biological and technical processes such as the anomalous diffusion in cellular environments [1–2], the delivery of drugs [3], the formation of colloidal crystals [4–5], particle sorting [6], and directed self-assembly [7]. Nanofluidic systems in general are characterized by spatial distances in at least one dimension of less than 100 nm. This distance range interferes with several natural length scales of particle–surface interactions [8], such as the electrostatic interactions. The electrostatic interactions between charged objects and surfaces in a nanofluidic system decay approximately exponentially with separation and a characteristic length scale termed Debye length [9].

Experimentally, the gap-distance-dependent forces between two curved surfaces were studied in micro-rheology experiments [10–11] and in detail using the surface force apparatus [12]. However, so far, most nanofluidic experiments involving confined particles have been performed using static surfaces and fixed geometries, which do not allow the degree of confinement to be varied in situ.

Recently it was demonstrated that the gap-distance-dependent electrostatic forces can be exploited to achieve geometry-induced trapping and manipulation of charged nanoparticles and vesicles in nanofluidic systems [13]. In a follow-up experiment, it was shown that crucial information on the trapping potential can be gained by using an AFM-type system and a micro-capillary to adjust the gap distance [14].

Another example of a strongly gap-dependent behavior is the lateral diffusion of particles in a nanofludic gap. In microfluidic systems, it has been shown that the theoretical predictions of hydrodynamically hindered diffusion are in agreement with the measured diffusivity of microparticles [15–16]. However, in nanofluidic systems, a 50–70% lower diffusion is observed when geometrical dimensions approach the Debye screening length [17–19]. The mechanisms that have been proposed to explain the increased hindrance are anomalous viscosity [17], anomalous diffusion [19] and an electroviscous effect [18].

Here we present a versatile setup that allows the distance between two parallel confining surfaces for samples of choice and a cover glass to be adjusted and measured with nanometer accuracy. First, we describe and characterize the system, and then demonstrate its utility by measuring the behavior of 60 nm charged Au nanospheres in confinement between a glass and a polymer surface. We first determine the height of the particles as a function of gap distance by means of their varying optical contrast. Next we determine the lateral diffusion for a range of fixed gap distances. The gap-dependent measurement allows us not only to measure the decreasing diffusion coefficients but also to determine the onset of a scale dependent diffusion induced by the roughness of the confining surfaces. A comparison with theory indicates that hydrodynamic effects alone cannot explain the behavior observed.

## Materials and Methods

### Nanofluidic confinement apparatus

**Figure 1:**
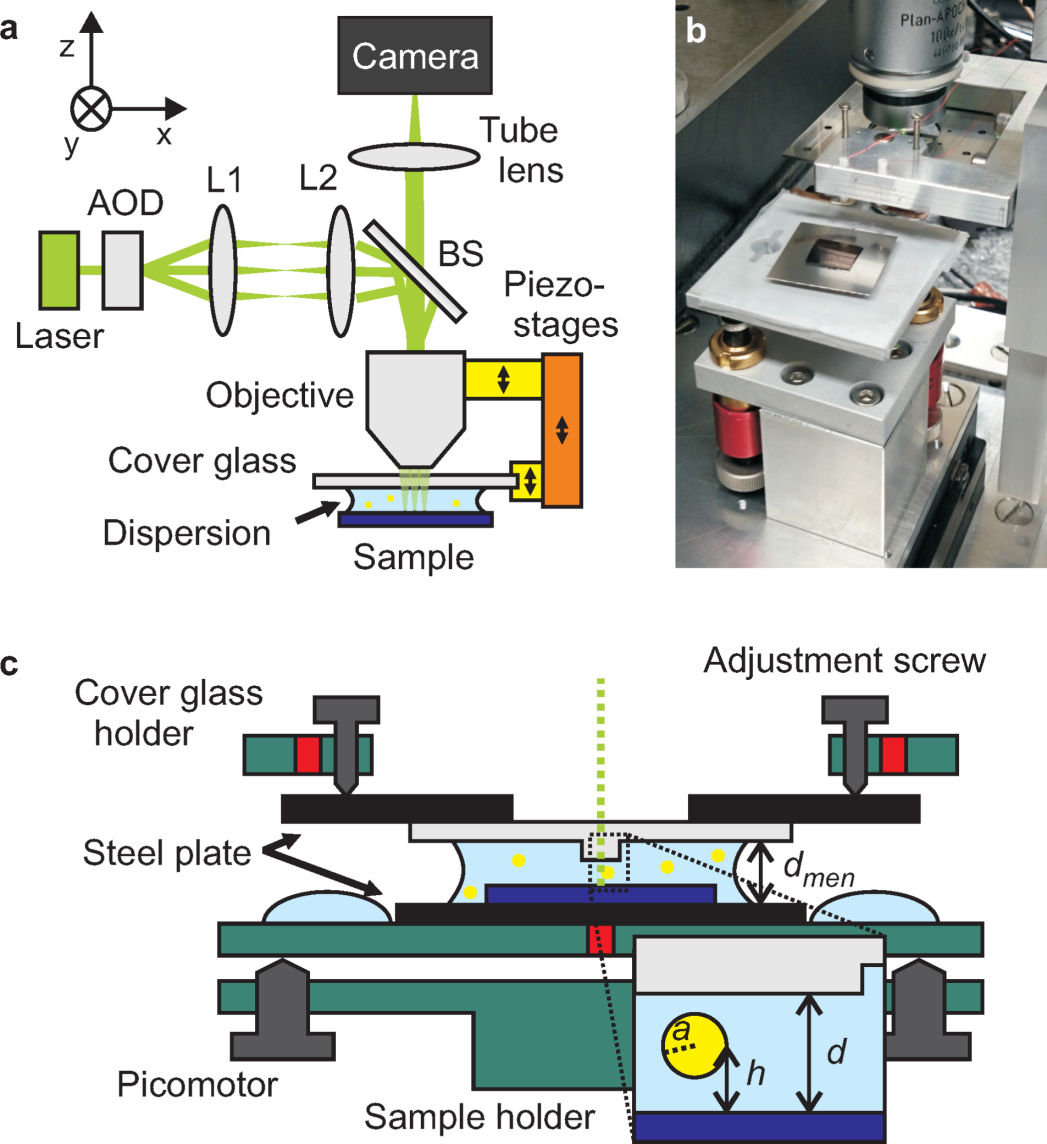
(a) The optical setup consist of a laser, an acousto-optic deflector (AOD), and a telecentric system (L1 and L2) for scanned laser illumination of the sample through a beam splitter (BS), and an oil immersion objective. Two linear piezo-stages (yellow) and one coarse-positioning stage (orange) allow to fine tune the focus and the confinement independently and to access a large range of gap distances. (b) Photograph of the nanofluidic confinement apparatus. (c) Sketch of the vertical profile of the system, see text for details. The inset visualizes the nanofludic slit with gap distance *d* confining a particle with radius *a* at height *h*.

A schematic illustration of the nanofluidic confinement apparatus is shown in Figure 1a. The optical illumination and detection scheme is based on interferometric scatterning detection (iSCAT) and was described in detail elsewhere [20–23], here we just provide a brief description.

By raster scanning the focus of a 532 nm continuous-wave laser (Samba 50 mW, Cobolt), the sample area of interest is illuminated. Scanning and focusing are done by a two-axis acousto-optic deflector (DTSXY, AA Opto-Electronic), a telecentric system, and a 100×, 1.4 numerical aperture (NA) oil-immersion objective (Alpha Plan-Apochromat, Zeiss). The reflected light is collected by the same objective, and images are captured by a high-frame-rate camera (MV-D1024-160-CL-12, Photon Focus). Typically we use a field of view of 300 × 300 pixels, corresponding to an area of 33 × 33 μm^2^. The imaging rate is 800 frames per second (FPS), given by the exposure time of 0.75 ms and a trigger delay of 0.5 ms, which was selected to avoid frame drops. We achieve uniform illumination using a single scan per frame and a laser line spacing of 500 nm, which is consistent with the measured laser spot size of ≈1 μm (measured at 1/e intensity).

The mechanical part with the tunable confinement setup is mounted below the objective (see Figure 1b). A schematic cross section through the center of the system is sketched in Figure 1c (not to scale): A droplet of particle dispersion is confined by the cover glass (light gray) and the sample (dark blue). The glass and the sample are both glued to steel plates (black). Magnets (red) in the aluminum holders (green) fix the position of the steel plates. Three adjustment screws are used to align the tilt of the cover glass with respect to the focal plane of the objective. Parallelization of the substrate to the cover glass is done by three linear piezo actuators (Picomotor, Newport). The distance of the cover glass and the microscope objective relative to the substrate is controlled by two linear piezo-stages (100 μm, Nano-OP100, Mad City Labs), which are attached to a coarse-positioning stage (MT-84, Feinmess). A 150–200 μm wide, square shaped mesa is etched in the cover glass such that the area outside the mesa is recessed by ≈50 μm (see next section for details). The mesa provides good optical access to the nanofluidic region and ensures that the gap distance *d* between the cover glass and sample (see inset of Figure 1c) can be reduced until a colloid has intimate contact to both surfaces.

A droplet volume of *V*_drop_ ≥ 20 μL is required such that the dispersion overflows the sample and wets the metal holder. This geometry increases the distance at the meniscus *d*_men_ to approximately 600 μm (sample thickness 550 μm). Therefore also the radius of curvature of the droplet is increased, resulting in a reduced Young–Laplace pressure and a high stability of the system. A water reservoir next to the the central droplet (Figure 1c) reduces the evaporation of the droplet in the slit and ensures system stability for several hours.

### Cover glass and sample preparation

The mesa of the cover glass (D263T borosilicate, UQG) was fabricated as follows: First, a masking layer of 30 nm Cr and 300 nm Au was sputtered onto the glass. Second, a photoresist (AZ4533, MicroChemicals) was spin coated and patterned by photolithography. Third, the masking layer was removed by wet etching (TechniEtch ACI2, MicroChemicals and TechniStrip Cr01, MicroChemicals) of the unprotected areas, leaving behind a central metal-resist stack defining the position of the mesa. The area around the stack was etched for 75 s by concentrated hydrofluoric acid (49% HF) to define the mesa. A mesa height of 40–45 μm was measured with a profilometer (Dektak, Veeco), corresponding to an etch rate of ≈36 μm/min, similar to the rate observed by Zhu et al. [24]. Finally, the remaining masking layer was removed by etching, and the processed cover glass was cleaned by peeling off a polymer layer (Red First Contact, Photonic Cleaning Technologies), in a helium plasma (Piezobrush, Relyon Plasma) for 20 s and by rinsing with ultrapure water (Millipore, 18 MΩ cm).

A 52 ± 1 nm thick cross-linking polymer (HM8006, JSR) was spin coated onto a silicon sample to increase adhesion for the subsequently spin coated 175 ± 2 nm thick poly-phthalaldehyde (PPA) film. The thicknesses were measured with AFM. The refractive indices *n*_HM_ = 1.67 and *n*_PPA_ = 1.59 were measured by ellipsometry. The surface potential of PPA in 1 mM KCl solution (pH 7–7.5) was measured in a Malvern Zetasizer to be −52 mV. A colloid of citrate stabilized 60 nm Au nanospheres (BBI Solutions) with a manufacturer-specified diameter of 2*a* = 59.8 ± 4.8 nm and density of ≈2.6 × 10^12^ particles per mL was diluted 1:10 in fresh ultrapure water (Millipore, 18 MΩ cm) to reduce the ion concentration. The diluted dispersion was used within a few hours. A pH of 6.8 ± 0.2, a zeta potential of ζ = −58 mV, a specific conductivity of Λ = 11.5 μS cm^−1^, and hydrodynamic diameter of 2*a* = 62.1 nm were measured for a 1:150 diluted dispersion using a Malvern Zetasizer. We observed a linear dependency between the conductivity and the degree of dilution, which is expected for strong electrolytes such as sodium citrate and sodium chloride. Both can be present, since the synthesis involves the reduction of chloroauric acid (HAuCl_4_) by sodium citrate (Na_3_Cit) [25]. The citrate also functions as a capping agent, therefore we first determine the cation concentration from the conductivity measurement and then estimate an upper limit for the Debye length of κ^−1^ ≈ 8.9 nm for the 1:10 diluted colloid. In an independent measurement, we determined a larger Debye length for the same but more diluted colloid, consistent with the Debye length presented here [23].

### Measurement of gap distance and stability of the mechanical setup

The performance of the setup is characterized by the precision achieved in controlling and detecting the gap distance. For a slit filled with aqueous dispersion, a change in gap distance leads to a change in the Young–Laplace pressure, which bends the cover glass such that the motion of the piezo and the cover glass are not in 1:1 correspondence. Therefore, we use the interference of the light between the sample and the cover glass as a measurement of the gap distance.

**Figure 2:**
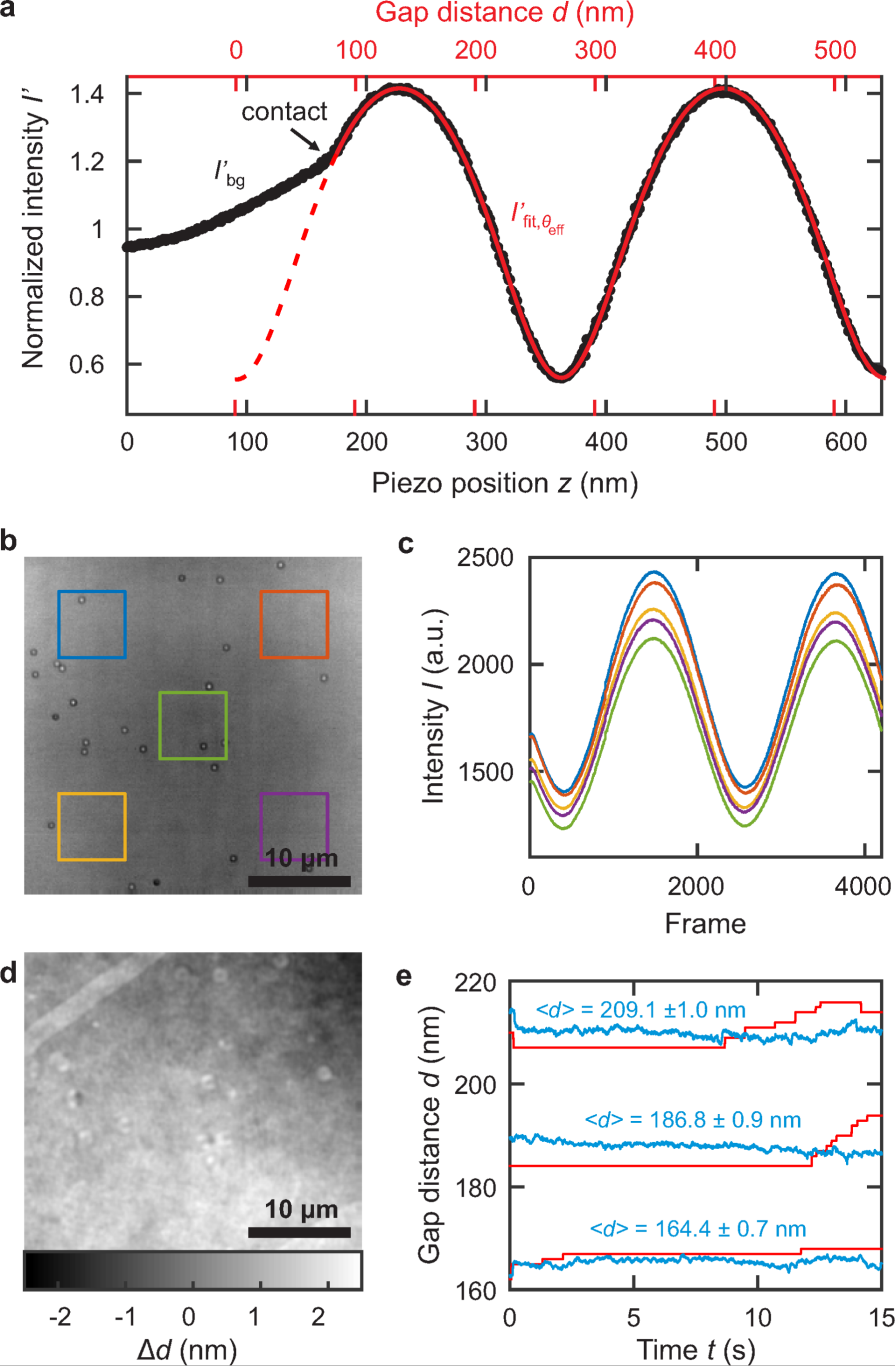
(a) Measured intensity reflected from a glass–air–silicon slit (black) while the cover glass is displaced vertically with a piezo motor. The red line depicts the result of a fit to our optical model. (b) Typical raw image of 60 nm Au nanospheres in the nanofluidc slit. (c) The median intensity captured in the areas indicated by the boxes in (b), while increasing the gap distance by 1 nm every 10th frame. (d) Effective gap distance variation Δ*d* in the nanofluidic slit obtained from the local variation in optical path difference. (e) The height of the cover glass (red) is adjusted by a feedback loop to ensure a constant gap distance (blue) during experiments.

For this measurement, we have to consider light rays departing from normal incidence, because we use a high NA objective to focus and collect the light. We address this issue by determining an effective incident angle as described in detail in [23]. The angle is determined from a measurement of the normalized interference intensity *I*′ as a function of the cover glass position *z* in air to avoid the effect of the pressure changes mentioned above, see Figure 2a. The signal arises from the interference of light rays reflected by the interfaces of the glass–water–polymer–silicon stack. We have developed an optical model [23] based on the transfer-matrix method, that considers the focusing of a Gaussian laser-beam. The result of a fit to the data is shown as red dashed line in Figure 2a. Fit parameters are the effective incident angle Θ_eff_ = 5.9° and the phase of the signal. The phase of the signal and the first contact point at a gap distance of *d* ≈ 80 nm fixes the absolute gap distance (see red axis). The required refractive indices for silicon, *n*_Si_ = 4.14, and for the cover glass, *n*_D263_ = 1.53, are taken from literature. To measure the gap distance in the water-filled system, we use the optical model and propagate the effective incident angle into the dielectric layers by using Snell's law.

Parallelization of the surfaces is achieved by measuring the interference signal in the four corners and at the center of the illuminated area (see Figure 2b): From the relative phase shift of the respective signals (see Figure 2c), the tilt of the confining surfaces can be determined. By tilting the sample, the phase difference was minimized using the cross-correlation of the corner to the center signals.

The optical path difference between glass and substrate varies because of the inherent surface roughness of the contributing interfaces. This fact leads to a varying phase shift of the interference signal pixel by pixel. AFM measurements yield the following root-mean-square (RMS) roughnesses: 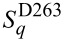 ≈ 0.4 nm for the cover glass, 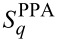 ≈ 0.3 nm for the polymer surface and 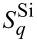 ≈ 0.2 nm for the silicon wafer. Since the silicon wafer is relatively flat and the refractive indices of polymer and glass are similar we approximate that all the phase differences originate from a roughness in the cover glass. The conversion from the phase shift to the gap distance is performed using the optical model mentioned above. The resulting gap distance image Figure 2d reveals a remnant tilt between the two confining surfaces, which could be corrected further. Without this correction, we achieve a height difference of 3 nm over a distance of 30 μm. The standard deviation of the plane corrected gap distance image is 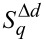 ≈ 0.6 nm, which is consistent with the measured surface roughness values.

During the measurements described in the subsequent sections, thermal drift and pressure changes may lead to a deflection of the relatively compliant cover glass. These deflections are compensated by implementing a closed-loop system, that registers changes in the background interference intensity and adjusts the height of the cover glass to keep the intensity constant. The feedback-loop can also operate during acquisition with a frequency of 20 Hz as illustrated by the red lines in Figure 2e. The blue lines indicate the measured laterally averaged gap distances for 15 s. The gap distance can be stabilized to ≈1 nm (1σ).

The stability of the setup in lateral direction was measured by observing the drift of a surface defect in the tool. A lateral drift of ≈100 nm/h was observed. We note that this drift is slow compared to the observed particle motion and does not affect the measurements. Another potential source for errors is squeeze-flow in the gap when modulating the gap distance. This effect can be observed by watching the particle motion during a fast approach motion of the two surfaces. In the center of the pillar this squeeze flow is minimal for symmetry reasons and we conducted the experiments at this location. Upon changing the gap distance to a new value we observed that the squeeze flow vanishes within a few seconds. Furthermore, the high stability of 1 nm of the gap distance during the diffusion measurements ensures that squeeze flow does not significantly contribute to the results.

### Particle localization

Radial symmetry-based tracking was used to identify the central lateral position of the nanosphere. This tracking algorithm yields similar accuracies compared to Gaussian fitting, is fast in execution, and detects any radially symmetric intensity distribution [26]. In particular the latter is important to detect the position at interference conditions for which the particle contrast vanishes at the center and only a diffraction ring of finite intensity is measured. We estimate an average lateral localization precision of ≤5 nm from the scatter of 35,000 detected positions obtained from 7 immobilized particles. This precision is in agreement with simulated particles [26] with a similar signal-to-noise ratio (SNR) of ≈20. We like to point out that we measure the same SNR using raw images similar to that in Figure 2b, but for moving particles we can reduce the fixed-pattern camera pixel noise of the background by subtracting the temporal median of the image stack. With that correction, we obtain a SNR of ≈50, which would correspond to a localization accuracy of less than σ*_i_* = 1.5 nm [26].

In order to assess the localization accuracy for a diffusing particle we have to consider our laser scanning illumination scheme using a focussed laser spot with σ*_L_* = 500 nm, as described above. Accordingly, a single point on the sample is scanned by ≈4 laser lines, separated by 500 nm and 10 μs, corresponding to a total time of τ_avg_ 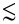 30 μs between the first and the forth scan line. During τ_avg_ the diffusion of a 60 nm Au nanoparticle (bulk diffusivity *D_p_* ≈ 7 μm^2^ s^−1^) in one dimension is 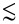

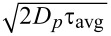
 ≈ 20 nm, which is small compared to the laser line spacing. Thus camera records an average image of the diffusing particle by integrating snapshots of the particle position when each laser scan line hits the particle at times *t_n_*. We may calculate an uncertainty σ*_d_* for the particle position assuming that the position is a simple weighted average of the individual particle positions. The weights *w_n_* are given by the normalized relative illumination for each scan line according to the Gaussian laser profile. Thus, σ*_d_* = 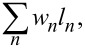 where *l_n_* = 
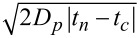
 is the diffusion length from the position when the beam hits the particle at its center at *t_c_*. Taking the uncertainty σ*_i_* for a fixed particle into account via error propagation, we obtain a localization accuracy of σ*_d_* ≈ 10 nm for the diffusing particles.

## Confined Lateral Diffusion

In the following we first revisit briefly the existing hydrodynamic models describing confined lateral particle diffusion. According to these models, the diffusivity depends not only on the gap distance but also on the vertical position of the particles in the gap. To test these predictions, we included in our measurements described in the subsequent sections not only the diffusion but also the height of the particles in the gap.

### Hydrodynamic models

Following the work of Eichmann et al. [18], we present the linear superposition (LSA) and the coherent superposition approximation (CSA) to calculate the hindered lateral diffusion in a fluidic slit. A third approximation, the matched asymptotic expansion (MAE), is not considered here as it deviates only slightly from the LSA.

The diffusion coefficient of a freely moving spherical particle obeys the Stokes–Einstein-equation

[1]
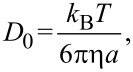


where *k*_B_ is Boltzmann’s constant, *T* is the absolute temperature, and η is the dynamic viscosity of the continuous medium. The hydrodynamically hindered diffusion parallel to a single interface is conveniently given by a correction factor *f*_||1_:

[2]
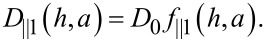


Solutions are given in terms of the dimensionless particle height, ω = *h*/*a*, for [27]

[3]
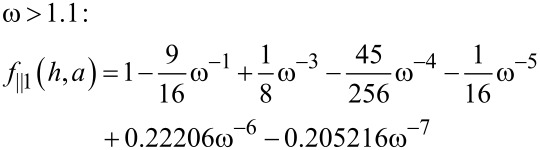


[4]
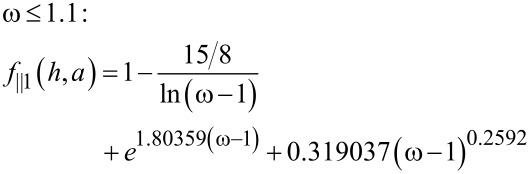


by Faxèn [28] and Goldman [29], respectively. A similar approach leads to the drag-reduced diffusion in a slit [30]:

[5]
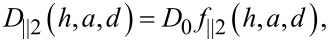


where *d* is the gap distance of the confining walls. Oseen suggested the LSA [30]

[6]
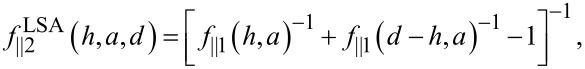


where the drag of each wall is treated independently and the total force is given by the sum of the contributions.

Anoher expression, the CSA

[7]
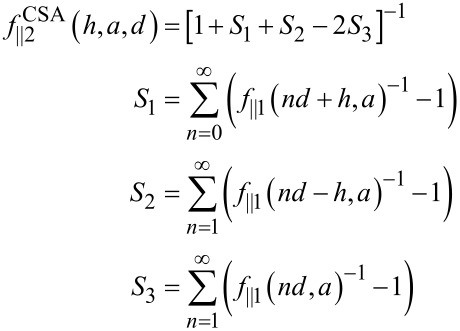


includes multiple interactions of the perturbations of the pressure and velocity fields induced by each wall. The same interactions with the colloid are not included [15,31].

The lateral diffusion coefficient *D*_||2_ can be measured from the mean squared displacement (MSD) in one of the orthogonal directions *x* or *y*. For the *x*-direction and a time interval Δ*t*, the MSD is given by

[8]
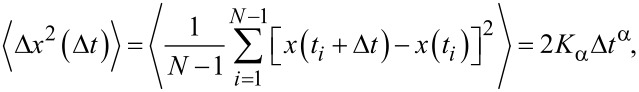


where 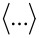 signifies the ensemble average, *N* is the number of observed positions per trajectory, *K*_α_ is a generalized diffusion coefficient and α is the anomalous diffusion exponent [32]. For α = 1, *K*_α_ corresponds to the lateral diffusion coefficient *D*_||2_, however, for 0 *<* α *<* 1 the behavior becomes subdiffusive. This situation is best described by a time-scale-depended diffusion coefficient *D_||2,α_*(Δ*t*) = *K*_α_Δ*t*^α−1^.

## Results and Discussion

### Particle height in an asymmetric slit

**Figure 3:**
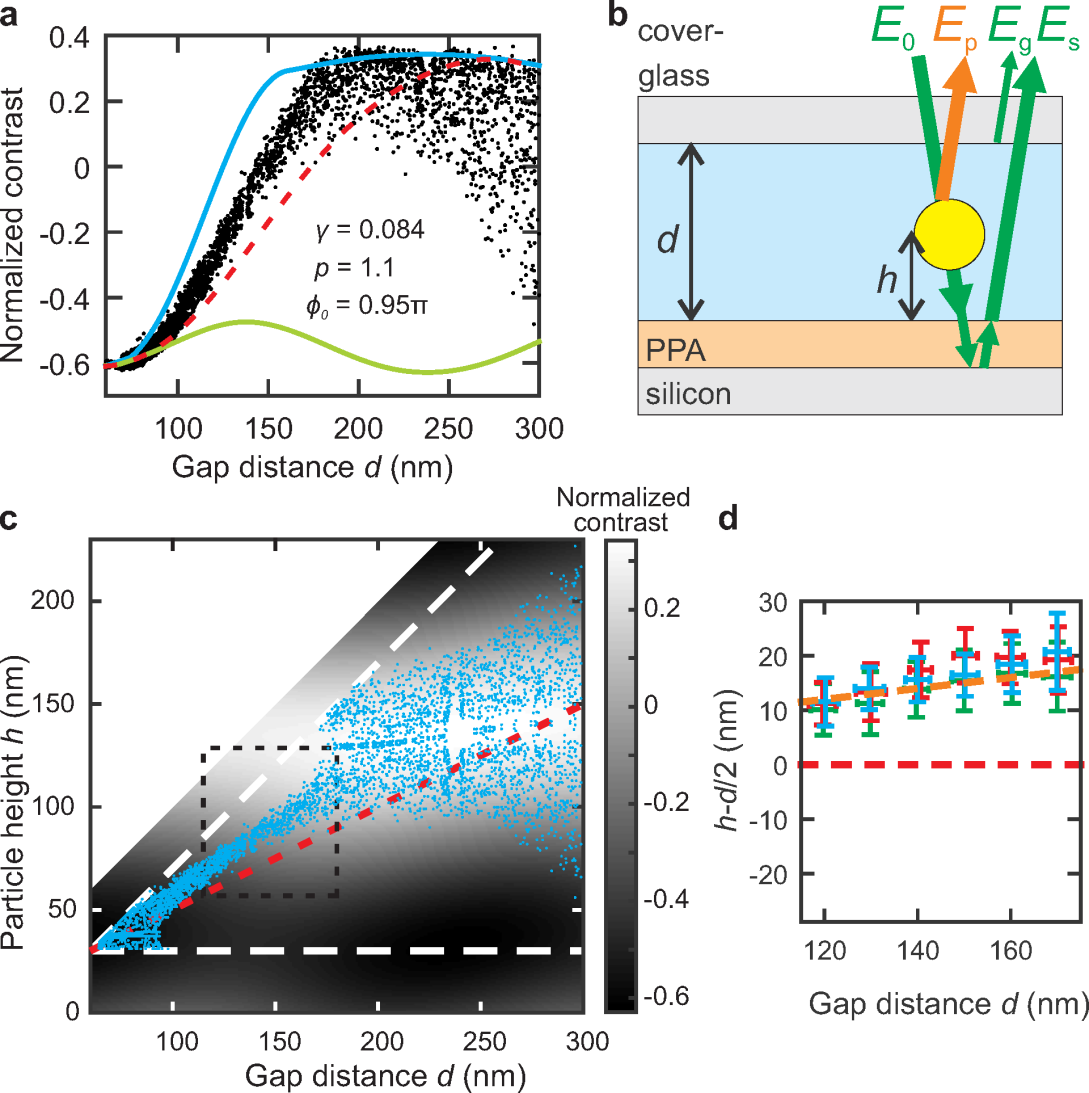
(a) Contrast signal of a nanosphere (dots), a simulated particle in the middle of the gap (dashed red line) and envelopes of simulated maximum (blue line) and minimum (green line) contrast for all possible particle heights. (b) Schematic illustration of the particle at height *h* in a gap of size *d*. Incoming laser light *E*_0_ is scattered by the particle (*E*_p_), partially transmitted and reflected at the substrate (*E*_s_) and reflected by the cover glass–water surface (*E*_g_). (c) Attributed particle heights (blue) are obtained by matching the measured (see panel a) and simulated contrast values (gray-scale image). The confining surfaces and the particle radius restrict the possible *h* values (dashed white lines). The dashed red line indicates the height values corresponding to the center of the gap. (d) Deviation of the observed particle height from the gap center *h* − *d*/2 for a gap distance range indicated by the dashed black box in panel c for three individually measured particles (blue, red, and green). The error bars indicate the standard deviation of *d* and *h*. Phenomenologically, the relative particle height follows *h*/*d* ≈ 0.61 (orange dashed line).

According to Equation 2–Equation 7, the height *h* of the particles influences the magnitude of the hindered diffusion. To quantify the effect, we first determine the height for an individually diffusing particle from its contrast. The scatter plot in Figure 3a depicts the experimentally measured and normalized contrast of such a particle for varying gap distance *d*.

The height of the particle in the gap relates to the intensity contrast of the particle with respect to the background that is observed in iSCAT imaging (see methods). For a fixed gap distance a sinusoidal dependence of the particle contrast with particle height was suggested [13]. The effect arises from the interference of the light scattered by the particle *E*_p_ with the background reflection, that is, the light reflected from the glass *E*_g_ and polymer/silicon interface *E*_s_, see Figure 3b. As discussed in the methods section, the background reflection is also a function of the gap distance, resulting in a more complex relation of the particle contrast with gap distance. In a previous publication we showed how the effective incident angle model describing the background reflection is extended to include the particle refection using three additional parameters to include the light scattered by a nanosphere in the nanofludic gap [23]. The first and the second parameter, *p* and 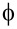
_0_, describe the amplitude and the accumulated phase of light scattered by the particle and collected by the camera. In addition, at the particle position, the light reflected by the substrate is reduced by a fraction γ. Due to the interferometric origin, the contrast of the particle is still a periodic function of the particle height with a period of ω_L_/2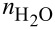 ≈ 200 nm, where ω_L_ = 532 nm is the laser wavelength and 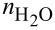 = 1.33 is the refractive index of water. In the experiments, we adjusted the polymer thickness to position the minimum of the particle contrast at tight confinement of *d* ≈ 70 nm, see Figure 3a. Consequently, a diffusing particle will probe the entire envelope of the contrast signal if it probes more than 100 nm of the height space above the minimum contrast position. In Figure 3a the black scatter plot indeed does not rise above a particle contrast of ≈0.35 and shows a turnaround at a particle contrast of ≈−0.6. Using the optical model described in detail in [23] the parameters γ, *p* and 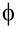
_0_ are iteratively optimized until the envelope predicted by the model (blue and green line in Figure 3a) matches the observed extremal contrast values, considering the finite range of possible particle heights given by the gap distance *d* and the finite radius *a* of the particle (*a* ≤ *h* ≤ *d*−*a*). The procedure ensures that the three parameters can be obtained without the need of additional height calibration using immobilized particles [23]. The red line illustrates the modeled contrast of a particle positioned in the middle of the gap.

The contrast modeled as a function of gap distance *d* and particle height *h* is shown as grayscale background in Figure 3c. To obtain the height values (blue dots) for a measured contrast we use the simulated values for a given gap distance as a lookup table. The short illumination time of 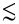
30 μs is essential to obtain reliable height-distribution data [33]. Nevertheless, similar to the lateral case, we expect an error of the vertical position accuracy for freely diffusing particles of approximately 10 nm. Consequently, the finite illumination time of 30 μs will reduce the width of the measured height distributions by several nanometers, depending on the steepness of the particle potential. The periodicity of the contrast signal with particle height leads to either one or multiple possible solutions for the particle height. In the single-value range of 115 nm ≤ *d* ≤ 175 nm we determined the averaged deviation *h* − *d*/2 of the particle height from the gap center (see Figure 3d).

Physically, the average height of the negatively charged particles is determined by the relative repulsion of the particles from the like charged confining surfaces. A height above the center of the gap indicates a higher charge on the polymer surface, which does not contain sites that could dissociate. However, it is known that hydrophobic surfaces often attain a negative charge in contact with water, most likely due to the preferential absorption of oxianions [34].

### Confined lateral diffusion of nanospheres

To measure the lateral diffusion of nanoparticles as a function of gap distance, we exploit the high mechanical stability and tunability of the nanofluidic confinement apparatus. We vary the gap distance for different measurements and then use the feedback-control loop to keep it constant (see Figure 2e) while acquiring frames for 15 s. The number of particles in the field of view reduces with decreasing gap distance. In our experiments at small distances the diffusion of at least 5 particles was measured. The high frame rate (800 FPS) nevertheless provides a sampling of 60,000 up to 300,000 particle positions for each measurement.

**Figure 4:**
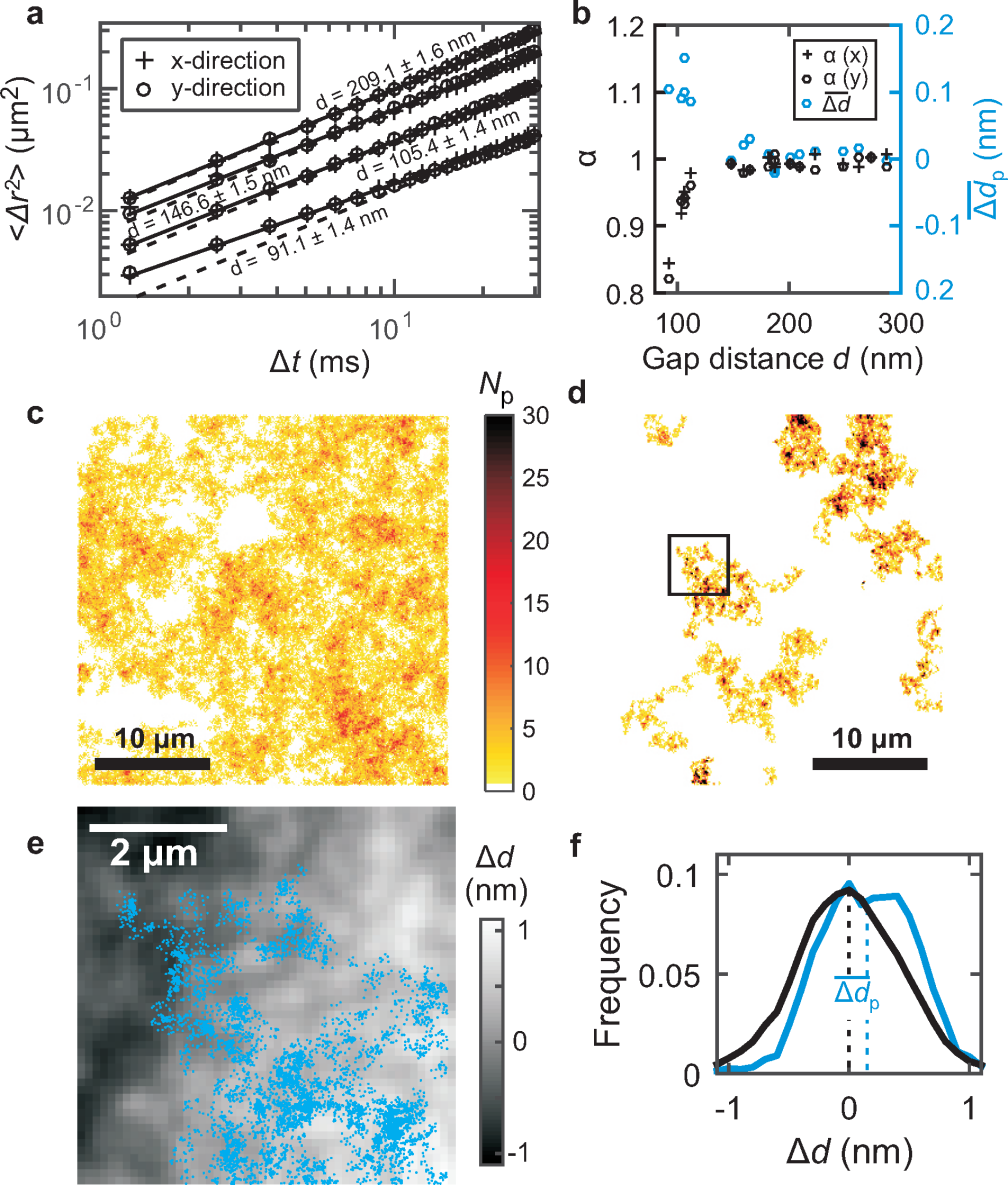
(a) Measurements of the lateral MSD in the *x*- and *y*-direction at four different gap distances *d*. The solid and the dashed lines indicate fits of Equation 8 to the data for anomalous and normal diffusion, respectively. (b) The parameter α indicates the degree of normal diffusion α = 1 and subdiffusion α *<* 1 (black symbols). An average value of the 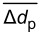 *>* 0 (see panel f) indicates that particles avoid narrow gap regions (blue circles). (c,d) Number of detected particles *N*_p_ in a 100 nm grid during a 15 s measurement at an average gap distance of (c) *d* = 210.0 ± 1.0 nm and (d) *d* = 105.9 ± 1.0 nm. (e) Gap distance modulation Δ*d* and detected particle positions (blue dots) for the area indicated by the black box in panel d. (f) Histograms of the gap distance modulation (black line) and for the locations of the gap distance modulation sampled by the particles (blue line).

For each gap distance *d*, we obtain the time and ensemble averaged MSD along *x*- and *y*-direction for a range of time steps Δ*t* from 1.25 ≤ Δ*t* ≤ 31.25 ms, see Figure 4a. A strong decrease of the diffusivity with decreasing gap distance is apparent. Fits of Equation 8 to the MSD in the *x*- and *y*-directions are given as solid lines. The dashed lines indicate fits to the data for normal diffusion (α = 1). The fit parameter α indicating subdiffusion for α *<* 1 is shown in Figure 4b. At a confinement *d <* 120 nm, a scale-dependent diffusion coefficient is observed, see also the increasing deviation of the dashed and solid lines in Figure 4a. This effect has been attributed to the presence of lateral obstacles preventing a free diffusion of the particles [35]. In our case, however, these obstacles are either induced by local charge inhomogeneities or by the roughness of the confining walls.

We use a simple picture to assess this hypothesis. In the so called linear superposition approximation the interaction energy *U*(*h*) of a charged spherical particle at a distance *h* to a charged plane is given by [23,36]:

[9]
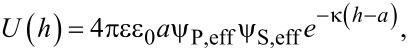


where κ^−1^ is the Debye length, ε is the dielectric constant of the medium, ε_0_ is the vacuum permittivity, *a* is the radius, and ψ_P,eff_ and ψ_S,eff_ are the effective surface potentials of plane and sphere, respectively. In this linear approximation the overall interaction energy of a sphere between two walls is obtained by the sum of the interaction energies to each wall. Assuming a surface potential of the sphere of −58 mV (see methods) and a surface potential of the walls of −67 mV as determined in our previous experiments [23], we obtain a change in interaction energy of ≈0.8*k*_B_*T* for a gap distance of 120 nm and a gap distance modulation of 1 nm. The simple model corroborates the interpretation that the observed RMS roughness of the glass of 0.4 nm provides significant energy barriers for diffusion. We note, however, that the same effect could be induced by a charge modulation of the surface potential (or correspondingly the surface charge) by ≈5%.

To further investigate the origin of the obstacles we analyzed the time-averaged lateral particle distribution and its correlation to the measured locally resolved gap distance variation Δ*d* (see Figure 2d). To obtain a measure for the particle distribution, we divide the field of view into a 100 nm grid and count the total number of particles visiting each grid section for all frames. The resulting number of detected particles is visualized as ”heatmaps” in Figure 4c,d for an average gap distance of (c) *d* = 209.1 ± 1.0 nm and (d) *d* = 105.4 ± 1.0 nm. The particles are quasi uniformly distributed over the entire field of view for the larger separation and are more localized in the narrower slit, leading to the observed higher local particle counts.

In order to correlate the detected particle trajectories with the gap distance modulation Δ*d*, see Figure 2d, we have to compensate for the tilt in the gap distance map. We divide the map into squares of 5 × 5 μm^2^ size, roughly corresponding to the diffusion length in *x*- and *y*-direction during the measurement of *r*_diff_ ≈ 5 μm, and correct for the offset in local gap distance modulation Δ*d* for each square. For example, Figure 4e shows Δ*d* and the positions of a single diffusing particle (blue dots) for the square given by the box in Figure 4d. According to this trace the particle samples certain locations of the Δ*d* map and we term the range of sampled values Δ*d*_p_. The average histograms of Δ*d* and Δ*d*_p_ for all squares are shown in Figure 4f as black and blue lines, respectively. Clearly, the particles prefer to be located at a position having a larger gap distance as apparent by the shift of the Δ*d*_p_ histogram to more positive Δ*d* values. To obtain a qualitative measure of the strength of this effect, we determined the distance of the center of mass of the two histograms 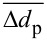 for all measured gap distances, see Figure 4f. The result is given in Figure 4b by the blue circles. For gap distances below *d* = 120 nm, a significant shift of the particle position into high-gap-distance positions is apparent. This behavior is qualitatively similar to the onset of subdiffusion measured for the MSD. Therefore, we conclude that the subdiffusion is indeed caused by the fact that the particles start to avoid regions with narrower gap distances.

**Figure 5:**
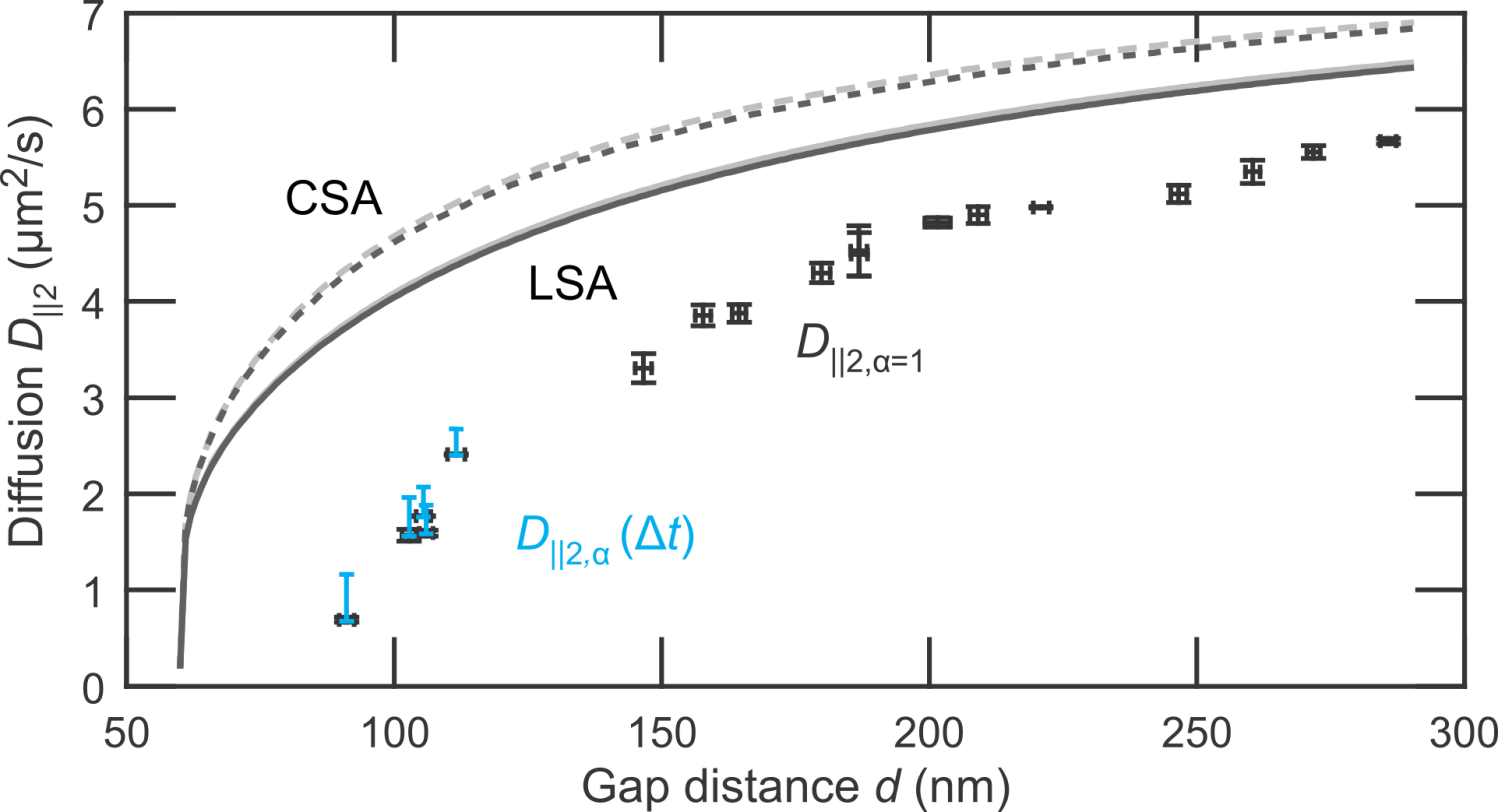
Lateral diffusion coefficients for varying gap distance in a nanofludic slit. The error of the normal diffusion coefficients *D*_||2_ (black scatter plot) and *d* are determined by the difference of *D*_||2_ in *x*- and *y*-direction and the standard deviation of *d*, respectively. The range of time dependent diffusion coefficients *D*_||2,α_(Δ*t*) for 1.25 ms *<* Δ*t <* 31.25 ms is plotted for *d <* 120 nm (blue bars). For greater gap distances the range of *D_||2,α_*(Δ*t*) is less than 8% of the normal diffusion coefficient. Theoretically predicted diffusion coefficients by LSA (solid lines, Equation 6) and CSA (dashed lines, Equation 7) are shown for an average particle height at *h* = 0.5*d* (gray) and *h* = 0.61*d* (black).

Now we turn to the central result, the gap-distance-dependent lateral diffusion coefficient *D*_||2_(*d*), which is depicted in Figure 5. The black scatter plot indicates the values for normal diffusion *D_||2,α=1_*(*d*) corresponding to the dashed lines in Figure 4a. For *d <* 120 nm subdiffusion is significant and a single diffusion coefficient is not sufficient to describe the process, see Equation 8. Instead, the diffusion coefficient *D_||2,α_*(*d*,Δ*t*) becomes dependent on the time interval Δ*t*. The range for *D_||2,α_*(*d*,Δ*t*) for 1.25 ms *<* Δ*t <* 31.25 ms is indicated for *d <* 120 nm by the blue bars.

For comparison, the predicted diffusion coefficients accounting for hydrodynamic hindrance from two walls are shown for the LSA (Equation 6 (solid lines)) and CSA (Equation 7 (dashed lines)). Both approximations were calculated for a particle diffusing at a measured height *h* = 0.61*d* (black) and in the middle of the slit *h* = 0.5*d* (gray). The asymmetric height leads to merely 1.5% lower diffusion coefficients and cannot explain the 20–50% lower diffusivity measured. We also exclude that the localization due to surface roughness is the predominant factor for this reduction, because pronounced subdiffusion is only observed for gap distances of *d <* 120 nm.

In bulk, the electroviscous effect is attributed to the surface charge of the particles and leads to an increased effective viscosity and thus to a reduction in particle diffusion [37]. A similar mechanism should also play a role in a nanofluidic system, in particular when a particle is close to a charged wall. Whereas diffusion measurements for uncharged particles [15] and for particles in electrolyte with higher ionic concentration [33] are in agreement with predictions that consider only a hydrodynamically hindered drag. There is considerable evidence of an increased drag of charged particles near charged walls in a weak electrolyte [18,38]. In a similar experimental configuration Eichmann et al. [18] measured a ≈30% (≈55%) lower lateral diffusion coefficient for 60 nm (100 nm) gold nanospheres with a relative radius of κ*a* ≈ 0.9 (κ*a* ≈ 2.1) and a relative glass-particle distance of κ*h* − κ*a* ≈ 4.5 (κ*h* − κ*a* ≈ 3.6). These values are in agreement with the ≈45% lower diffusion we measure for κ*a* ≈ 3.4 and κ*h* − κ*a* ≈ 4.

## Conclusion

We have developed a new versatile setup for investigating the behavior of nano-objects in a tunable confinement between two surfaces. The interferometric detection setup allows us not only to detect the nano-objects with high sensitivity, but also to determine the 3D particle position and the wall separation in situ with nanometer spatial and millisecond temporal precision. Furthermore, a diffraction limited resolved map of the sub-nanometer-resolved gap distance can be obtained. We use the tool to measure the height and diffusion of 60 nm gold spheres as a function of absolute gap distance between a glass and a polymer surface. We find that the particles localize more closely to the glass interface indicating a higher charge of the polymer surface. Subdiffusion becomes significant at gap distances below *d* = 120 nm. We demonstrate that this scale dependent diffusion is correlated to particle trajectories that avoid regions of narrow gap distances caused by the surface roughness of the confining surfaces. The measured lateral diffusion coefficients are 20–50% lower than predicted by purely hydro-dynamical hindrance, also when taking their asymmetric position in the gap into account. Similarly, the observed scale dependent diffusion cannot account for the effect because it is only significant for small gap distances. We conclude that electro-viscous effects are the main cause for the observed reduction in diffusivity. Our measurements provide a detailed information on the gap-distance-dependent particle diffusion, which may form the basis for testing theories describing the electro-viscous effect. In general, the results shown here demonstrate the versatility of the tool which allows one to measure nanoparticle behavior as a function of confinement in remarkable detail.

## References

[1] Regner B M, Vučinić D, Domnisoru C, Bartol T M, Hetzer M W, Tartakovsky D M, Sejnowski T J (2013). Biophys J.

[2] Baum M, Erdel F, Wachsmuth M, Rippe K (2014). Nat Commun.

[3] Langer R, Peppas N A (2003). AIChE J.

[4] Gong T, Wu D T, Marr D W M (2002). Langmuir.

[5] Reinmüller A, Oğuz E C, Messina R, Löwen H, Schöpe H J, Palberg T (2012). J Chem Phys.

[6] Huang L R, Cox E C, Austin R H, Sturm J C (2004). Science.

[7] Grzelczak M, Vermant J, Furst E M, Liz-Marzán L M (2010). ACS Nano.

[8] Bocquet L, Tabeling P (2014). Lab Chip.

[9] Hunter R J, White L R (1987). Foundations of colloid science.

[10] Dhinojwala A, Granick S (1997). J Chem Phys.

[11] Clasen C, McKinley G H (2004). J Non-Newtonian Fluid Mech.

[12] Israelachvili J N (1992). Intermolecular and Surface Forces.

[13] Krishnan M, Mojarad N, Kukura P, Sandoghdar V (2010). Nature.

[14] Kim J T, Spindler S, Sandoghdar V (2014). Nat Commun.

[15] Lin B, Yu J, Rice S A (2000). Phys Rev E.

[16] Dufresne E R, Altman D, Grier D G (2001). Europhys Lett.

[17] Kaji N, Ogawa R, Oki A, Horiike Y, Tokeshi M, Baba Y (2006). Anal Bioanal Chem.

[18] Eichmann S L, Anekal S G, Bevan M A (2008). Langmuir.

[19] Zhao L, Zhong Y, Wei Y, Ortiz N, Chen F, Wang G (2016). Anal Chem.

[20] Jacobsen V, Stoller P, Brunner C, Vogel V, Sandoghdar V (2006). Opt Express.

[21] Kukura P, Ewers H, Müller C, Renn A, Helenius A, Sandoghdar V (2009). Nat Methods.

[22] Mojarad N, Sandoghdar V, Krishnan M (2013). Opt Express.

[23] Fringes S, Skaug M, Knoll A W (2016). J Appl Phys.

[24] Zhu H, Holl M, Ray T, Bhushan S, Meldrum D R (2009). J Micromech Microeng.

[25] Frens G (1973). Nature (London), Phys Sci.

[26] Parthasarathy R (2012). Nat Methods.

[27] Pawar Y, Anderson J L (1993). Ind Eng Chem Res.

[28] Faxèn H (1923). Ark Mat, Astron Fys.

[29] Goldman A J, Cox R G, Brenner H (1967). Chem Eng Sci.

[30] Oseen C W (1927). Neuere Methoden und Ergebnisse in der Hydrodynamik.

[31] Lobry L, Ostrowsky N (1996). Phys Rev B.

[32] Metzler R, Klafter J (2000). Phys Rep.

[33] Eichmann S L, Bevan M A (2010). Langmuir.

[34] Tian C S, Shen Y R (2009). Proc Natl Acad Sci U S A.

[35] Volpe G, Volpe G, Gigan S (2014). Sci Rep.

[36] Adamczyk Z, Warszyński P (1996). Adv Colloid Interface Sci.

[37] Conway B, Dobry-Duclaux A, Eirich F (1960). Rheology: Theory and Applications.

[38] Carbajal-Tinoco M D, Cruz de León G, Arauz-Lara J L (1997). Phys Rev E.

